# Progression of chronic wasting disease in white-tailed deer analyzed by serial biopsy RT-QuIC and immunohistochemistry

**DOI:** 10.1371/journal.pone.0228327

**Published:** 2020-02-14

**Authors:** Davin M. Henderson, Nathaniel D. Denkers, Clare E. Hoover, Erin E. McNulty, Sarah K. Cooper, Lauren A. Bracchi, Candace K. Mathiason, Edward A. Hoover

**Affiliations:** Department of Microbiology, Immunology, and Pathology, Prion Research Center, College of Veterinary Medicine and Biomedical Sciences, Colorado State University, Fort Collins, Colorado, United states of America; National Institute of Allergy and Infectious Diseases, UNITED STATES

## Abstract

Chronic wasting disease (CWD) continues to spread or be recognized in the United States, Canada, and Europe. CWD is diagnosed by demonstration of the causative misfolded prion protein (PrP^CWD^) in either brain or lymphoid tissue using immunodetection methods, with immunohistochemistry (IHC) recognized as the gold standard. In recent years, *in vitro* amplification assays have been developed that can detect CWD prion seeding activity in tissues, excreta, and body fluids of affected cervids. These methods potentially offer earlier and more facile detection of CWD, both pre- and post-mortem. Here we provide a longitudinal profile of CWD infection progression, as assessed by both real-time quaking-induced conversion (RT-QuIC) and IHC on serial biopsies of mucosal lymphoid tissues of white-tailed deer orally exposed to low doses of CWD prions. We report that detection of CWD infection by RT-QuIC preceded that by IHC in both tonsil and recto-anal lymphoid tissue (RAMALT) in 14 of 19 deer (74%). Of the 322 biopsy samples collected in post-exposure longitudinal monitoring, positive RT-QuIC results were obtained for 146 samples, 91 of which (62%) were concurrently also IHC-positive. The lower frequency of IHC positivity was manifest most in the earlier post-exposure periods and in biopsies in which lymphoid follicles were not detected. For all deer in which RT-QuIC seeding activity was detected in a tonsil or RAMALT biopsy, PrP^CWD^ was subsequently or concurrently detected by IHC. Overall, this study (a) provides a longitudinal profile of CWD infection in deer after low yet infectious oral prion exposure; (b) illustrates the value of RT-QuIC for sensitive detection of CWD; and (c) demonstrates an ultimate high degree of correlation between RT-QuIC and IHC positivity as CWD infection progresses.

## Introduction

Chronic wasting disease (CWD) is an emergent, fatal, neurological prion disease affecting deer, elk, reindeer and moose in the United States, Canada, the Republic of Korea and all Scandinavian countries [1–6]. CWD is unique as a prion disease in its capacity to spread in free-ranging and captive cervid populations despite concentrated control efforts [7–9].

Rapid, sensitive detection of CWD in live cervids is important given the long pre-symptomatic, yet prion shedding phase of the disease and the uncertain risks posed by the consumption and transport of sub-clinically infected animals. Sensitive CWD detection is especially pertinent to cervids with polymorphism changes in the prion protein gene in which disease course progression is prolonged [10–12].

The gold standard for CWD diagnosis is detection of the abnormal, misfolded prion protein (PrP^RES/CWD^) in the brain stem or retropharyngeal lymph node by immunohistochemistry (IHC) or enzyme-linked immunosorbent assay (ELISA) [13, 14]. In the last decade, the development of amplification methods—notably, serial protein misfolding cyclic amplification (sPMCA) and real-time quaking induced conversion (RT-QuIC)—have vastly increased CWD prion detection sensitivity [15–18].

We and others have used RT-QuIC to detect and estimate levels of prion seeding activity in multiple tissues, excreta, and body fluids with the goal of increasing sensitivity and speed in CWD detection pre- and post-mortem [19–26]. Here we provide a dynamic longitudinal profile of progressing CWD infection in white-tailed deer after low dose oral exposure to CWD prions as assessed by serial lymphoid tissue biopsies examined by both the RT-QuIC and IHC methods.

## Results

### Detection of CWD seeding activity precedes detection of PrP^CWD^ deposition by IHC

**Fig 1:**
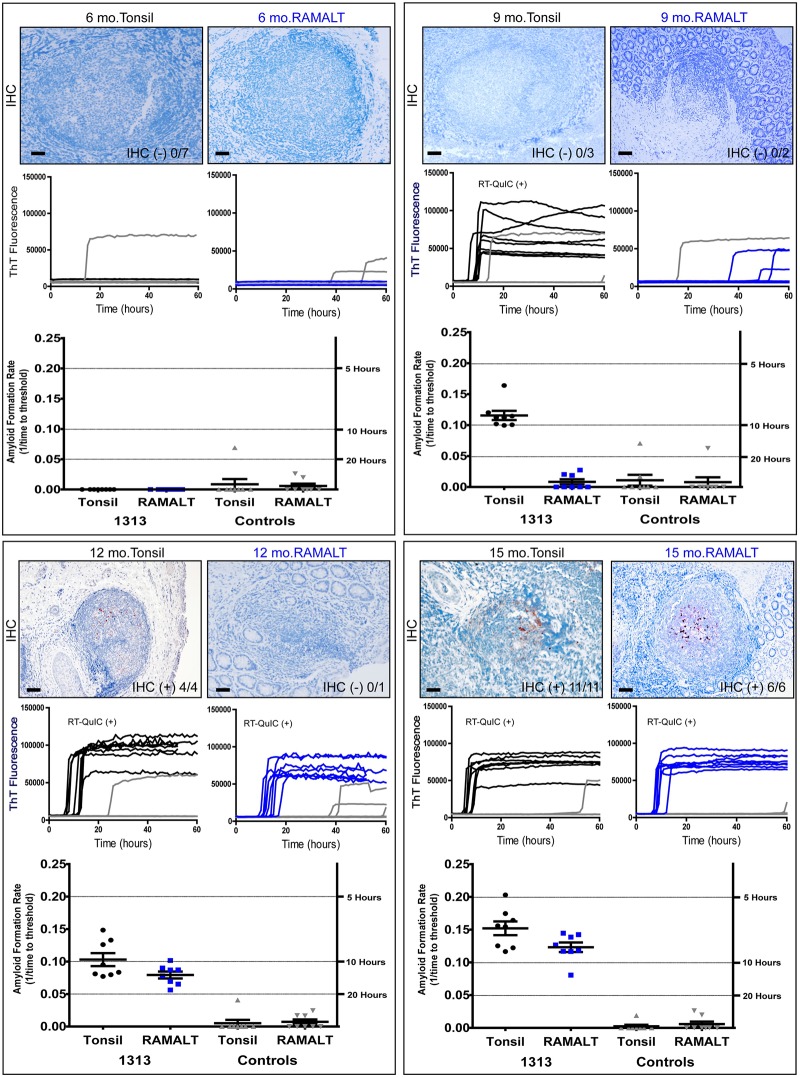
RT-QuIC seeding activity precedes IHC detection of PrP^CWD^ in tonsil and RAMALT biopsies. Six, nine, twelve and fifteen-month biopsy samples of tonsil (black) and RAMALT (blue) were probed with anti-PrP antibody BAR224 for PrP^CWD^ detection by IHC. IHC immunoreactivity is denoted in the bottom right corner of the image with number of positive follicles in the sample over the total follicle count. Scale bar is 100 μM except for the 15-month tonsil image which is 50 μM. RT-QuIC ThT traces are displayed below the corresponding IHC time-point. Negative (grey) replicates are also shown. Each RT-QuIC reaction was performed twice with 4 replicates per experiment. Positive RT-QuIC experiments are noted in each panel as determined by MW-test. Reaction rate data with matched controls are shown below the raw data traces.

**Fig 2:**
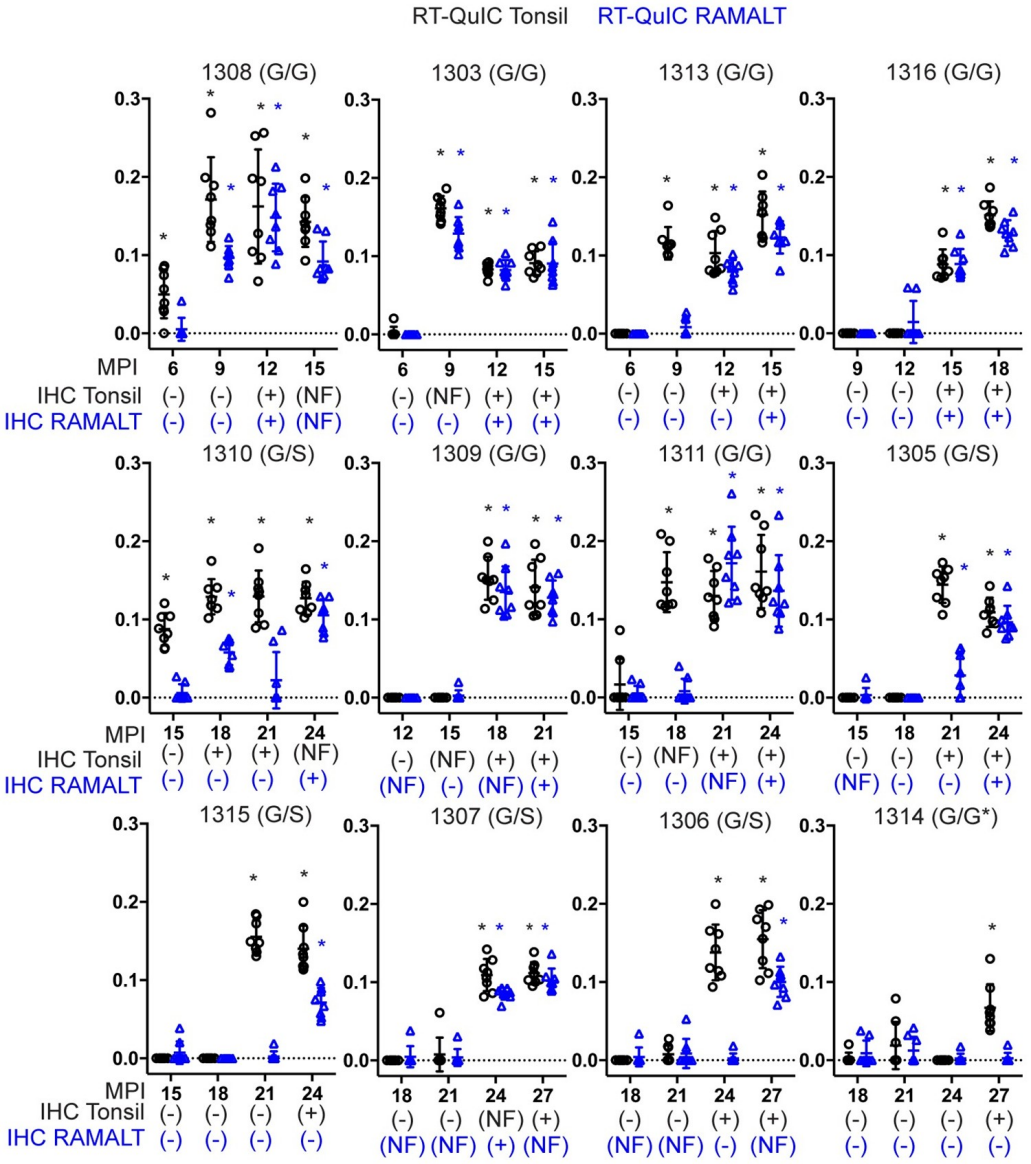
Comparison of IHC and RT-QuIC detection in tonsil and RAMALT biopsies. Each plot displays a 12-month timeframe where deer became IHC positive for the first time. Months post inoculation is on the x-axis and RT-QuIC reaction rate is displayed on the y-axis. The IHC status of each deer is displayed for tonsil and RAMALT biopsies below each timepoint as positive negative or no follicle (NF). The deer number and genotype are displayed on top of each graph. Tonsil RT-QuIC and IHC data is in black. RAMALT RT-QuIC and IHC data is in blue. RT-QuIC tests that are deemed positive by MW-test are marked with a star.

We compared RT-QuIC and IHC to detect CWD infection in longitudinal studies of white-tailed deer cohorts orally exposed to low concentrations of CWD prions (10mg, 1mg, or 300ng CWD-positive brain or equivalent CWD(+) saliva) over a multi-year disease course. In serial biopsies from deer (n = 12) exposed to either 1mg or 300ng of CWD(+) material, we detected RT-QuIC seeding activity earlier than IHC detection of PrP^CWD^ deposition in 15 (7 tonsil, 8 RAMALT) of 24 biopsies (62.5%) (Figs 1 and 2; Tables 1 and 2). Fig 1 depicts a typical longitudinal RT-QuIC and IHC profile in a single deer.

**Table 1:** Complete results for low dose inoculation group. Results for RT-QuIC, NAPTA RT-QuIC and IHC for 6 months through 30 months are presented. RT-QuIC data are presented as the number of positive replicates over total. RT-QuIC results that were statistically significant by MW-test are in red. IHC results are reported as follicles with positive staining over total follicles. If at least one follicle had positive staining the result was considered positive and is in red. Biopsies with no follicles present are reported as no follicle (NF). Samples for which no remaining homogenate was available for testing are reported as no sample (NS). Cross symbols indicate the animal was deceased at the collection time point. In most cases, after two IHC positive biopsies, no samples were collected from that point forward and indicated as did not collect (DNC).

	Animal #	1308	1303	1313	1316	1310	1309	1311	1305	1315	1307	1306	1314
	Dose	1mg	300ng	300ng	300ng	1mg	300ng	300ng	1mg	300ng	300ng	300ng	1mg
	96 Genotype	G/G	G/G	G/G	G/G	G/S	G/G	G/G	G/S	G/S	G/S	G/S	G/G
**6 Months (Mo)**	QuIC (+) T	7/8	1/8	0/8	1/8	1/8	0/8	0/8	1/8	1/8	0/8	2/8	1/8
	QuIC (+) R	1/8	0/8	0/8	3/8	0/8	0/8	0/8	0/8	0/8	0/8	0/8	2/8
	NAPTA QuIC (+) T	NS	1/8	4/8	NS	4/8	1/8	3/8	NS	1/8	3/8	5/8	4/8
	NAPTA QuIC (+) R	2/8	0/8	3/8	6/8	4/8	0/8	4/8	1/8	2/8	0/8	3/8	2/8
	IHC T (+)	0/5	0/3	0/7	0/3	0/8	0/1	0/3	0/3	0/1	0/2	0/3	0/8
	IHC R (+)	0/2	0/6	0/22	0/3	0/5	0/3	0/3	0/2	0/5	0/5	0/2	0/5
**9 Mo**	QuIC (+) T	8/8	8/8	8/8	0/8	4/8	0/8	0/8	0/8	1/8	0/8	0/8	1/8
	QuIC (+) R	8/8	8/8	3/8	0/8	6/8	3/8	3/8	3/8	2/8	0/8	1/8	5/8
	NAPTA QuIC (+) T	8/8	7/8	5/8	2/8	NS	7/8	2/8	7/8	0/8	0/8	2/8	NS
	NAPTA QuIC (+) R	8/8	8/8	1/8	3/8	7/8	5/8	2/8	2/8	1/8	6/8	1/8	4/8
	IHC T (+)	0/2	NF	0/3	0/7	0/8	0/4	0/2	0/2	0/5	NF	0/7	0/6
	IHC R (+)	NF	0/3	0/2	0/7	0/8	NF	0/2	0/1	NF	0/4	0/2	0/8
**12 Mo**	QuIC (+) T	8/8	8/8	8/8	0/8	4/8	0/8	0/8	2/8	7/8	0/8	0/8	7/8
	QuIC (+) R	8/8	8/8	8/8	2/8	8/8	0/8	0/8	1/8	0/8	0/8	2/8	1/8
	NAPTA QuIC (+) T	8/8	NS	8/8	NS	5/8	2/8	7/8	2/8	NS	NS	1/8	4/8
	NAPTA QuIC (+) R	8/8	8/8	8/8	5/8	7/8	8/8	5/8	4/8	3/8	1/8	6/8	5/8
	IHC T (+)	2/2	9/9	2/4	0/3	NF	0/7	0/1	0/2	0/5	0/1	0/12	0/5
	IHC R (+)	3/3	2/2	0/1	0/10	0/4	NF	0/1	0/7	0/4	0/2	0/3	NF
**15 Mo**	QuIC (+) T	8/8	8/8	8/8	8/8	8/8	0/8	2/8	0/8	0/8	1/8	3/8	2/8
	QuIC (+) R	8/8	8/8	8/8	8/8	2/8	1/8	2/8	1/8	2/8	3/8	1/8	4/8
	NAPTA QuIC (+) T	8/8	NS	8/8	8/8	8/8	3/8	NS	5/8	3/8	6/8	4/8	8/8
	NAPTA QuIC (+) R	8/8	8/8	8/8	8/8	3/8	4/8	4/8	6/8	8/8	1/8	4/8	7/8
	IHC T (+)	NF	6/6	11/11	2/2	0/1	NF	0/1	0/2	0/7	0/3	0/10	0/2
	IHC R (+)	NF	2/2	6/6	2/5	0/4	0/4	0/4	NF	0/4	0/1	0/3	0/2
**18 Mo**	QuIC (+) T	DNC	8/8	DNC	8/8	8/8	8/8	8/8	0/8	0/8	0/8	0/8	1/8
	QuIC (+) R	DNC	8/8	DNC	8/8	8/8	8/8	2/8	0/8	0/8	1/8	1/8	2/8
	NAPTA QuIC (+) T	DNC	8/8	DNC	8/8	NS	8/8	8/8	3/8	4/8	NS	1/8	4/8
	NAPTA QuIC (+) R	DNC	8/8	DNC	8/8	8/8	8/8	3/8	2/8	8/8	2/8	3/8	6/8
	IHC T (+)	DNC	1/2	DNC	6/6	8/10	7/7	NF	0/9	0/8	0/12	0/3	0/2
	IHC R (+)	DNC	4/4	DNC	2/2	0/5	NF	0/5	0/3	0/1	NF	NF	0/5
**21 Mo**	QuIC (+) T	†	†	DNC	DNC	12/12	12/12	8/8	12/12	8/8	1/8	3/12	4/12
	QuIC (+) R	†	†	DNC	DNC	3/12	12/12	8/8	9/12	1/8	1/8	2/12	4/12
	NAPTA QuIC (+) T	†	†	DNC	DNC	8/8	8/8	8/8	8/8	8/8	6/8	2/8	3/8
	NAPTA QuIC (+) R	†	†	DNC	DNC	2/8	8/8	NS	6/8	4/8	7/8	0/8	4/8
	IHC T (+)	†	†	DNC	DNC	3/3	5/6	1/1	2/2	0/8	0/4	0/1	0/1
	IHC R (+)	†	†	DNC	DNC	0/1	1/1	NF	0/1	0/1	NF	0/8	0/5
**24 Mo**	QuIC (+) T	†	†	DNC	†	8/8	DNC	8/8	8/8	8/8	8/8	8/8	0/8
	QuIC (+) R	†	†	DNC	†	8/8	DNC	8/8	8/8	8/8	8/8	1/8	1/8
	NAPTA QuIC (+) T	†	†	DNC	†	8/8	DNC	8/8	8/8	8/8	8/8	8/8	3/8
	NAPTA QuIC (+) R	†	†	DNC	†	8/8	DNC	8/8	8/8	8/8	8/8	4/8	8/8
	IHC T (+)	†	†	DNC	†	NF	DNC	1/2	5/5	8/9	NF	6/8	0/2
	IHC R (+)	†	†	DNC	†	1/1	DNC	14/14	2/2	0/6	1/3	0/4	0/6
**27 Mo**	QuIC (+) T	†	†	†	†	DNC	DNC	DNC	DNC	8/8	8/8	8/8	8/8
	QuIC (+) R	†	†	†	†	DNC	DNC	DNC	DNC	1/8	8/8	8/8	1/8
	NAPTA QuIC (+) T	†	†	†	†	DNC	DNC	DNC	DNC	8/8	8/8	8/8	8/8
	NAPTA QuIC (+) R	†	†	†	†	DNC	DNC	DNC	DNC	3/8	8/8	8/8	2/8
	IHC T (+)	†	†	†	†	DNC	DNC	DNC	DNC	1/2	2/6	6/8	1/2
	IHC R (+)	†	†	†	†	DNC	DNC	DNC	DNC	NF	NF	NF	NF
**30 Mo**	QuIC (+) T	†	†	†	†	†	†	DNC	†	8/8	†	8/8	8/8
	QuIC (+) R	†	†	†	†	†	†	DNC	†	4/8	†	7/8	8/8
	NAPTA QuIC (+) T	†	†	†	†	†	†	DNC	†	8/8	†	8/8	8/8
	NAPTA QuIC (+) R	†	†	†	†	†	†	DNC	†	6/8	†	8/8	6/8
	IHC T (+)	†	†	†	†	†	†	DNC	†	13/13	†	3/3	8/12
	IHC R (+)	†	†	†	†	†	†	DNC	†	NF	†	0/1	0/11

**Table 2:** Time in months that RT-QuIC detected CWD seeding activity before PrP^CWD^ detected by IHC. The time in months prior to IHC detection for each deer is displayed. The average times span at which each test detected CWD is also displayed for all deer, 96GG deer and 96GS deer. A blank space indicates that the tissue was not found to be positive in the 30-month experimental span.

Low dose inoculation group								
Animal #	1308 (G/G)	1303 (G/G)	1313 (G/G)	1316 (G/G)	1309 (G/G)	1311 (G/G)	1314 (G/G)	Avg. (G/G)
RT-QuIC Tonsil	6	3	3	0	0	3	15	4.28
RT-QuIC RAMALT	3	3	3	0	3	3		2.50
NAPTA Tonsil	3	3	3	0	9	9	12	5.57
NAPTA RAMALT	3	3	3	9	12	0		5.0
Animal #	1310 (G/S)	1305 (G/S)	1315 (G/S)	1307 (G/S)	1306 (G/S)	Avg. (G/S)		Avg. all
RT-QuIC Tonsil	3	0	12	3	0	3.60		4
RT-QuIC RAMALT	15	3		0		6.00		3.66
NAPTA Tonsil	6	12	3	6	0	5.40		5.5
NAPTA RAMALT	15	9		15		13.00		7.66
10 mg inoculation group								
Animal #	1031 (G/G)	1076 (G/G)	1078 (G/G)	1079 (G/G)	1081 (G/G)	1082 (G/G)	1093 (G/G)	Avg.
RT-QuIC Tonsil	0	2	1	0	4	0	3	1.42
RT-QuIC RAMALT		0	1	0	0	0	3	0.67

CWD seeding activity was detected in tonsil biopsies an average of 4 months before detection of PrP^CWD^ by IHC (paired t-test, p = 0.0155). In RAMALT biopsies, RT-QuIC positivity preceded IHC positivity by an average of 3.66 months (paired t-test, p = 0.0384) (Tables 1 & 2). In all biopsies collected, the overall correlation between a positive RT-QuIC and a positive IHC result was 83%. However, for all deer in which RT-QuIC positivity was detected in a tonsil or RAMALT biopsy, PrP^CWD^ was subsequently detected by IHC in at least one, and usually both, of those tissues.

In the deer cohort (n = 8) orally exposed to 10 mg CWD(+) brain, RT-QuIC seeding activity preceded IHC detection in both tonsil and RAMALT biopsies in 4 of 7 deer (57%) (Table 3). RT-QuIC detection preceded IHC positivity by an average of 1.4 months in tonsil biopsies (paired t-test, p = 0.0582) and 0.67 months in RAMALT biopsies (paired t-test, p = 0.2354) (Table 2). Similar to the lower dose cohorts, all deer in which RT-QuIC positivity was detected subsequently became IHC-positive in tonsil and/or RAMALT biopsy.

**Table 3:** Summary of results for the higher dose group. Results for RT-QuIC and IHC for 3 months through 20 months are presented. RT-QuIC data is presented as the number of positive replicates over total. RT-QuIC results that were statistically significant by MW-test are in red. IHC results are reported as follicles with positive staining over total follicles. If at least one follicle had positive staining, the sample was considered positive and is in red. Biopsies that did not contain lymphoid follicles are reported as no follicle (NF). After two IHC positive biopsies, serial samples were not collected (DNC).

	Animal #	1031	1076	1078	1079	1081	1082	1093
	96 Genotype	G/G	G/G	G/G	G/G	G/G	G/G	G/G
3 Months	QuIC (+) T	0/8	8/8	2/8	0/8	2/8	1/8	8/8
	QuIC (+) R	2/8	0/8	0/8	0/8	2/8	0/8	8/8
	IHC (+) T	NF	0/1	NF	NF	0/1	NF	NF
	IHC (+) R	0/1	NF	0/12	0/2	0/1	0/1	0/4
5 months	QuIC (+) T	0/8	8/8	1/8	1/8	8/8	8/8	8/8
	QuIC (+) R	2/8	3/8	1/8	1/8	8/8	6/8	8/8
	IHC (+) T	NF	5/7	0/3	0/4	NF	1/3	0/1
	IHC (+) R	0/1	0/1	0/1	0/5	3/5	1/2	0/1
6 months	QuIC (+) T	1/8	8/8	4/8	0/8	8/8	8/8	7/8
	QuIC (+) R	1/8	8/8	4/8	1/8	8/8	8/8	8/8
	IHC (+) T	NF	4/6	NF	NF	NF	2/3	1/1
	IHC (+) R	0/2	4/5	0/6	0/2	NF	6/9	2/4
8 months	QuIC (+) T	1/8	8/8	6/8	0/8	4/8	8/8	8/8
	QuIC (+) R	1/8	8/8	5/8	0/8	8/8	8/8	8/8
	IHC (+) T	0/8	5/5	NF	0/4	NF	3/3	1/2
	IHC (+) R	0/1	38/40	NF	NF	1/1	NF	5/5
9 months	QuIC (+) T	0/8	8/8	8/8	0/8	8/8	8/8	8/8
	QuIC (+) R	0/8	8/8	8/8	0/8	8/8	8/8	8/8
	IHC (+) T	0/2	NF	4/5	NF	3/3	NF	1/1
	IHC (+) R	0/3	2/3	0/2	0/2	1/2	2/2	4/4
10 months	QuIC (+) T	0/8	8/8	8/8	1/8	8/8	8/8	8/8
	QuIC (+) R	0/8	8/8	8/8	0/8	8/8	8/8	8/8
	IHC (+) T	0/1	NF	NF	0/3	3/3	3/4	NF
	IHC (+) R	NF	2/3	3/4	0/2	0/1	NF	5/5
11 months	QuIC (+) T	0/8	7/8	8/8	0/8	8/8	8/8	8/8
	QuIC (+) R	0/8	8/8	8/8	0/8	8/8	8/8	8/8
	IHC (+) T	0/1	NF	0/1	0/2	NF	11/11	NF
	IHC (+) R	0/2	1/1	2/2	0/1	1/1	2/2	2/3
12 months	QuIC (+) T	0/8	8/8	8/8	0/8	8/8	8/8	8/8
	QuIC (+) R	0/8	8/8	8/8	0/8	8/8	8/8	8/8
	IHC (+) T	0/1	NF	1/1	0/3	5/5	6/6	5/5
	IHC (+) R	0/7	2/2	10/10	0/2	8/10	6/6	1/3
14 months	QuIC (+) T	0/8	8/8	8/8	0/8	8/8	8/8	8/8
	QuIC (+) R	1/8	8/8	8/8	0/8	8/8	8/8	8/8
	IHC (+) T	0/7	3/3	2/2	0/4	3/3	6/6	3/3
	IHC (+) R	0/1	3/3	NF	NF	1/1	2/2	NF
16 months	QuIC (+) T	0/8	DNC	DNC	0/8	DNC	DNC	DNC
	QuIC (+) R	1/8	DNC	DNC	0/8	DNC	DNC	DNC
	IHC (+) T	NF	DNC	DNC	NF	DNC	DNC	DNC
	IHC (+) R	NF	DNC	DNC	NF	DNC	DNC	DNC
18 months	QuIC (+) T	8/8	DNC	DNC	8/8	DNC	DNC	DNC
	QuIC (+) R	3/8	DNC	DNC	1/8	DNC	DNC	DNC
	IHC (+) T	2/3	DNC	DNC	1/2	DNC	DNC	DNC
	IHC (+) R	0/2	DNC	DNC	NF	DNC	DNC	DNC
20 months	QuIC (+) T	8/8	DNC	DNC	8/8	DNC	DNC	DNC
	QuIC (+) R	8/8	DNC	DNC	8/8	DNC	DNC	DNC
	IHC (+) T	NF	DNC	DNC	NF	DNC	DNC	DNC
	IHC (+) R	NF	DNC	DNC	3/5	DNC	DNC	DNC

### NaPTA-RT-QuIC can enhance CWD detection

**Fig 3:**
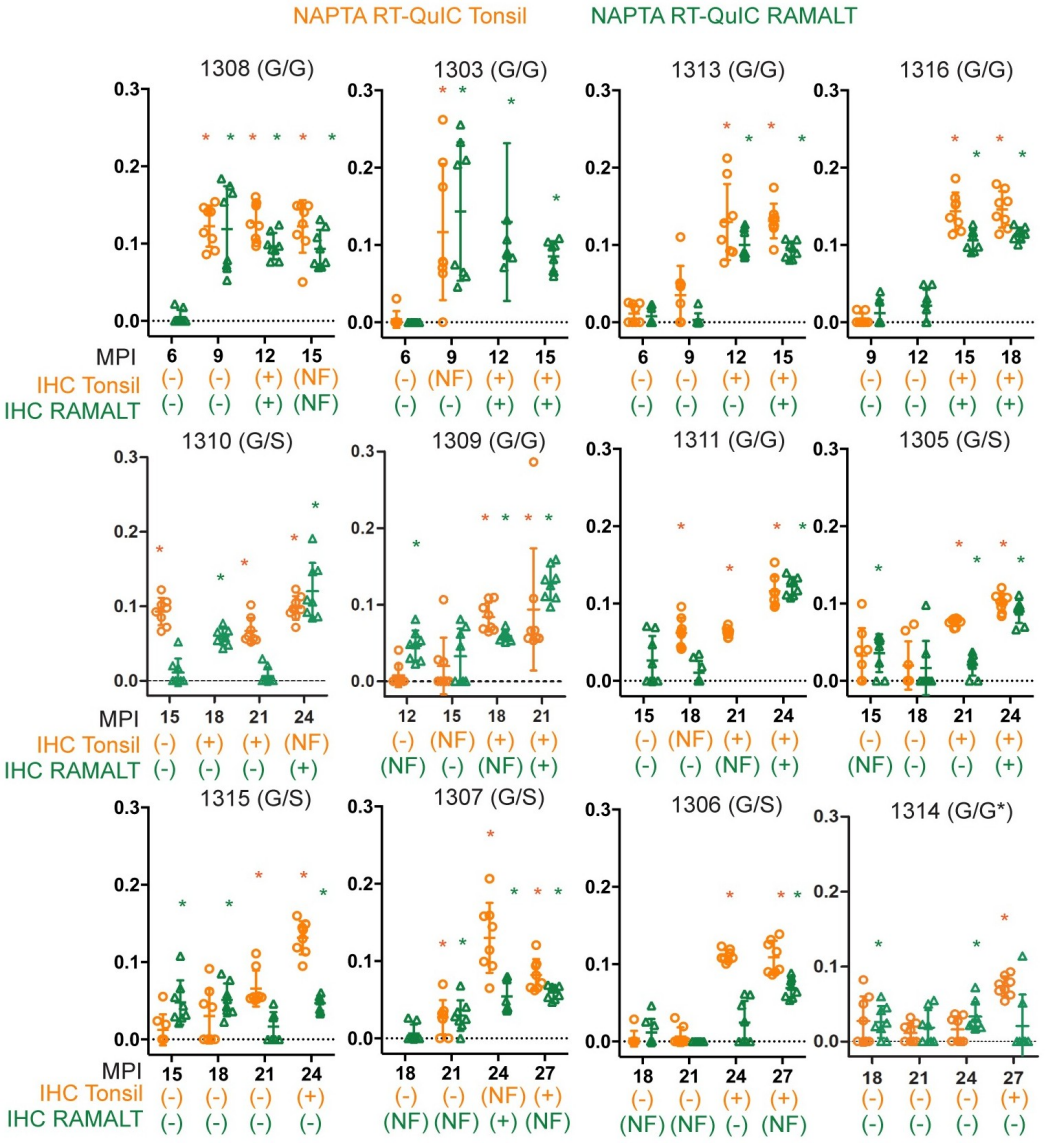
Comparison of IHC and NAPTA RT-QuIC detection in tonsil and RAMALT biopsies. Each plot displays a 12-month timeframe where deer became IHC positive for the first time. Months post inoculation is on the x-axis and RT-QuIC reaction rate is displayed on the y-axis. The IHC status of each deer is displayed for tonsil and RAMALT biopsies below each timepoint as positive negative or no follicle (NF). The deer number and genotype are displayed on top of each graph. Tonsil RT-QuIC and IHC data is in orange. RAMALT RT-QuIC and IHC data is in green. RT-QuIC tests that are deemed positive by MW-test are marked with a star.

NaPTA precipitation followed by RT-QuIC often enhanced CWD detection in tonsil and RAMALT biopsies in the lower dose cohorts (Fig 3, Table 2). Particularly in some 96GS genotype animals, NaPTA-QuIC produced positive results months earlier than did standard RT-QuIC, although variation in detection consistency among assay replicates also appeared to be somewhat greater (Table 2). Overall, longitudinal biopsy results indicated that RT-QuIC positivity presaged PrP^CWD^ detection by IHC. With passage of time, the two assays were highly correlated.

### Presence or absence of lymphoid follicles in biopsy samples

CWD detection in biopsies is heavily influenced by the presence or absence of lymphoid follicles. For example, in this study, circumstances forced the use of different biopsy instruments in the 10 mg dose cohort. Unfortunately, this led to 30 of 81 tonsil biopsies (37%) in which no lymphoid follicle could be detected by IHC examination. Whereas in the lower dose (1 mg and 300 ng) cohorts, in which the usual biopsy instrument was used, only 8 of 80 tonsil biopsies (10%) were without lymphoid follicles, as determined by IHC examination. Summing these study arms, a total of 72 biopsy samples (tonsil and RAMALT) lacked lymphoid follicles, and therefore no PrP^CWD^ aggregates were detected by IHC. The paired halves of the same 72 biopsy samples examined by RT-QuIC yielded 32 positives (44%) (Tables 1 & 3). Of the remaining 40 biopsies that did not have follicles, 20 were samples taken prior to any detection of a CWD infection and therefore may have been true negatives. As in all study cohorts, all IHC-positive tissue biopsies were also RT-QuIC positive. Thus, RT-QuIC appears to be more likely to detect CWD positivity in sub-optimal biopsy samples in which no lymphoid follicles have been detected.

### Overall comparison of IHC and RT-QuIC in CWD detection via lymphoid tissue biopsy

Over the duration of these studies, 322 longitudinal biopsies from 20 white-tailed deer were assayed by both RT-QuIC and IHC. CWD seeding activity was detected by RT-QuIC in 146 of 322 (45%) biopsies. PrP^CWD^ deposition was detected by IHC in 91 of these 322 (28%) biopsies. All 91 samples that were positive by IHC were also positive in RT-QuIC (Tables 1 and 3). Conversely, no IHC-positive/RT-QuIC negative sample were detected. The lower frequency of IHC positivity is influenced by the number of relatively early post-exposure samples in the study and the number of biopsies which did not contain lymphoid follicles. With passage of time, in all the 19 CWD-exposed deer that developed CWD infection, the agreement of the two assays was ultimately 100%.

## Discussion

This longitudinal analysis of tonsil and RAMALT biopsies by RT-QuIC and IHC demonstrated that detection of CWD by RT-QuIC preceded that by IHC by ~3.8 months (range: 0–15) in 14 of 19 deer (74%). We interpret this result to reflect greater sensitivity of RT-QuIC vs. IHC for early CWD detection. While this time span is usually small relative to the long course of CWD infection, its significance can be magnified when live animals are being tested to ensure against movement or importation of pre-symptomatic carriers. This latter advantage of RT-QuIC accompanies its greater assay speed, scale-ability, versatility, and potential for application to other clinically accessible samples from live animals and the environment [27–30].

The time span to first positive assay for the cohorts exposed to the lower prion doses (1mg or 300ng of CWD+ material) compared to the 10mg cohort was moderately lengthened—consistent with a minimum threshold achieved to trigger local amplification, progression, and pathogenesis. Ongoing studies to more closely define such threshold and any potential impact of serial exposure are needed to better define minimum dose and better understand CWD transmission in nature.

The present study reinforces current information indicating a slower progression of CWD in animals with PRNP genotypes 96GS, 96SS, and 95H [31–33], including delayed IHC detection of PrP^CWD^ in lymphoid tissues. This difference could reflect either slower conversion, leading to a higher degree of variability, or greater protease sensitivity of 96GS prions. With regards to the former, the difference in variability could be related to the small sample size and that two [2] deer never had an IHC-positive RAMALT. Larger samples sizes with additional genotype variants would help elucidate this issue. Relative to the latter, because RT-QuIC does not require fixation or protease digestion, it may be able to detect a wider array of prion seeds.

An issue presented in applying RT-QuIC to detect CWD in living, or even post-mortem animals, will be instances of RT-QuIC positive/IHC negative results. In the present study, all deer in which biopsies were positive in RT-QuIC subsequently became IHC positive ~3.8 months later. Of note, 2 tonsil samples (1315 & 1314; 12MPI) were extreme outliers becoming IHC positive 12 and 15 months later. While we speculated on human, sample, or assay error, we were unable to determine the cause of these results. Conversely, no IHC-positive/RT-QuIC negative results were obtained (Tables 1 and 3). Thus, RT-QuIC positivity portends IHC positivity in signifying CWD infection. Consistent with this tenet are the findings from RT-QuIC re-testing of elk in natural herds was employed [34] as do findings from analysis of prion seeding activity in nasal brush samples in Creutzfeldt-Jakob Disease patients and suspects [27].

Going forward, additional longitudinal studies on living animals using both assays are likely the most practical way to help address assay agreement issues. Likewise, bioassays or cervid cell panel in vitro infection assays [35] on such RT-QuIC+/IHC- samples will also be valuable. Continued improvements in RT-QuIC methodology, enrichment protocols [36, 37], and multi-laboratory confirmation assays on blinded sample panels will advance the use of amplification assays in CWD detection.

In summary, here we profile the progression of CWD in white-tailed deer after low dose oral exposure to CWD prions and demonstrate that RT-QuIC positivity in tonsil and rectal biopsies precedes and ultimately correlates with PrP^CWD^ deposition in paired lymphoid tissues. These results demonstrate the utility of real-time conversion for rapid sensitive detection of CWD infection in cervids.

## Materials and methods

### Inoculation cohorts and sampling

Deer studies were conducted at the indoor CWD research facility at Colorado State University under the strict guidelines approved by the USDA, NIH, and the Institutional Animal Care and Use Committee (Protocol Number: 18-8396A). White-tailed deer (*Odocoileus virginianus*) were provided at 5 months of age by the University of Georgia (Warnell School of Forestry and Natural Resources), which is a non-CWD endemic region. Deer were housed socially in suites (6×12 meter) with sand/epoxy mixture sealed floors (for normal hoof wear) and a layer of aspen chip bedding. Light cycles alternated every 12 hours, temperatures were kept between 16–26 degrees Celsius, and humidity was maintained at 25–40%. Ropes, toys, cardboard boxes, and daily interaction with caretakers were sources of enrichment. Deer received 50 g complete pelleted feed per kg/day along with hay forage and water ad libitum. Deer were anesthetized IM via dart with Ketamine (2–8 mg/kg) and Medetomidine (0.1–0.2 mg/kg) for sample collection (including prior to euthanasia) and euthanasia was performed by IV injection of pentobarbital sodium with phenytoin (1 ml per 4.5 kg).

Two [2] groups of deer in separate years were inoculated with either CWD (+) brain homogenate or saliva via the oral/*per os* (PO) route. PRNP genotypes at codon 96 (GG/GS) were determined prior to establishing cohorts in order to try and provide equal ratios. Group 1 (low-dose) comprised 3 cohorts that were inoculated as follows: Cohort 1 (n = 4) received 1 mg brain tissue as a single dose (deer 1305,1308,1310,1314); Cohort 2 (n = 4) received 3-100ng brain material doses over 3 weeks (deer 1303,1307, 1315, 1316); Cohort 3 (n = 4) received 3–10 ml saliva doses over 3 weeks (deer 1306,1309,1311,1313). Group 2 (high-dose) contained a single cohort (n = 8), which received 10mg brain tissue PO as a single dose. Longitudinal tonsil and RAMALT biopsies were collected from each cohort on individual days, within each suite, every 1–3 months and were analyzed for prion seeding activity or PrP^RES^ by either RT-QuIC or immunohistochemistry, respectively.

### IHC analysis of biopsy samples

IHC detection of PrP^RES^ was performed using a modified version of a previously described protocol [38]. Briefly, PLP-fixed tissues were paraffin embedded following standard histology procedures. 5 μm sections were placed on positive-charged glass slides and tissues were rehydrated through graded alcohol baths, treated with 88% formic acid for 30 min, then processed through a heat-induced epitope retrieval in citrate buffer. Endogenous peroxidase activity was quenched by treatment with 3% hydrogen peroxide and tissues were blocked prior to incubation overnight with anti-cervid prion protein mouse monoclonal antibody BAR-224 (1mg/mL; Caymen Chemical) diluted 1:750. Secondary detection was with anti-mouse Envision +System HRP (DAKO) and visualized with AEC substrate (ABCam). Negative control tonsil and RAMALT tissues were run simultaneously and showed no immunoreactivity.

### RT-QuIC methods

Protein substrate for RT-QuIC reactions was prepared as previously described [39]. Syrian hamster PrP^C^ residues 90–231 (SH90-231) was purified by on column refolding in Ni-sepharose fast flow resin (GE health sciences). A 180-milliliter linear gradient from 6M guanidine hydrochloride, 100 mM NaPO_4_, 10 mM tris base pH 8.0 to 100 mM NaPO_4_, 10 mM tris base pH 8.0 was used for on column refolding. Elution was achieved by a linear gradient of 100 mM NaPO_4_, 10 mM tris base pH 8.0 to 100 mM NaPO_4_, 10 mM tris base, 500 mM imidazole pH 8.0. Protein was dialyzed at 0.5 mg/ml in two changes of 4L 10 mM NaPO_4_, pH 5.5.

RT-QuIC conditions were 0.1 mg/ml PrP^C^ SH90-23, 100 mM NaPO_4_, 320 mM NaCl, pH 7.4. An SDS concentration of 0.002% was used for both dilution RT-QuIC and NAPTA RT-QuIC. Reactions were sealed in Greiner optical bottom black 96-well plates and placed in a BMG fluostar Omega plate reader. The plate reader was set to read every 15 minutes at a gain of 1700 and an excitation of 450 and emission of 480. In between fluorescence measurements plates were shaken for a minute every other minute at 700 RPMs in double orbital mode. For all experiments, the plate readers executed 250 cycles for an approximate reaction time of 62.5 hours. Reactions were considered positive if baseline fluorescence values exceeded 5 standard deviations from the initial fluorescence. We used a unpaired, non-parametric rank based test (Mann-Whitney test (MW-test)) to determine significance of RT-QuIC reaction rate values compared to negative controls (all at 62.5 hours), similar to those previously described [40]. Each plate contained negative control tonsil and RAMALT samples, run in quadruplicate, which were only compared to data within a single 96-well plate.

### RT-QuIC analysis of tonsil and RAMALT biopsy samples

Tonsil and RAMALT biopsies were homogenized in 1x phosphate buffered saline (PBS) pH 7.4 at an initial 10% weight/volume concentration. Samples were subsequently diluted to a final concentration of 10^−3^ in 0.1% SDS. Samples were analyzed in quadruplicate (2μL/well) on two separate plates to obtain at least 8 replicates, at a minimum. Test samples were compared to matched replicates [8] from CWD (-) tonsil and RAMALT samples from deer inoculated with CWD(-) material.

### NaPTA RT-QuIC analysis of biopsy samples

10 μL of a 10% tissue homogenate was diluted to 1% in 0.1% SDS/1xPBS. 100 μL of the 1% solution was incubated and shaken with 7 μL of sodium-phosphotungstic acid solution (0.5 g sodium phosphotungstic acid, 0.43 g MgCl_2_-6-hydrate, pH 4.0) on a thermomixer for 1 hour at 37 C. The solution was pelleted by centrifugation for 30 minutes at 15,000 rpm (21,130xg). The supernatant was removed, and the pellet resuspended in 10 μL 0.1% SDS/1xPBS. Two microliters of precipitated solution were used to seed each RT-QuIC well for a total of at least 8 replicates on two separate experiments. Test samples were compared to 8 replicates of CWD (-) submandibular lymph node and RAMALT samples from deer inoculated with CWD(-) material.

## Supporting information

S1 Checklist The ARRIVE guidelines checklist. (PDF)
